# Seeking for Non-Zinc-Binding MMP-2 Inhibitors: Synthesis, Biological Evaluation and Molecular Modelling Studies

**DOI:** 10.3390/ijms17101768

**Published:** 2016-10-22

**Authors:** Alessandra Ammazzalorso, Barbara De Filippis, Cristina Campestre, Antonio Laghezza, Alessandro Marrone, Rosa Amoroso, Paolo Tortorella, Mariangela Agamennone

**Affiliations:** 1 Dipartimento di Farmacia, Università “G. d’Annunzio” Chieti, Via dei Vestini 31, 66100 Chieti, Italy; alessandra.ammazzalorso@unich.it (A.A.); barbara.defilippis@unich.it (B.D.F.); cristina.campestre@unich.it (C.C.); alessandro.marrone@unich.it (A.M.); rosa.amoroso@unich.it (R.A.); 2 Dipartimento di Farmacia-Scienze del Farmaco, Università “A. Moro” Bari, Via Orabona 4, 70125 Bari, Italy; antonio.laghezza@uniba.it (A.L.); paolo.tortorella@uniba.it (P.T.)

**Keywords:** MMPs, inhibitors, molecular dynamics, docking, quinoline, isoquinoline, ureas, non-zinc-binding group

## Abstract

Matrix metalloproteinases (MMPs) are an important family of zinc-containing enzymes with a central role in many physiological and pathological processes. Although several MMP inhibitors have been synthesized over the years, none reached the market because of off-target effects, due to the presence of a zinc binding group in the inhibitor structure. To overcome this problem non-zinc-binding inhibitors (NZIs) have been recently designed. In a previous article, a virtual screening campaign identified some hydroxynaphtyridine and hydroxyquinoline as MMP-2 non-zinc-binding inhibitors. In the present work, simplified analogues of previously-identified hits have been synthesized and tested in enzyme inhibition assays. Docking and molecular dynamics studies were carried out to rationalize the activity data.

## 1. Introduction

Matrix metalloproteinases are calcium and zinc-containing proteases (24 in humans) involved in the hydrolysis of the extracellular matrix (ECM) components and, therefore, responsible for tissue remodeling in physiological conditions [1]. Unbalanced matrix metalloproteinase (MMP) activity has been observed in many pathologies, such as cancer, neurodegeneration, cardiovascular diseases and others [2,3,4,5,6,7,8,9,10,11]; for this reason, they represent a well-studied therapeutic target. More recently, it has been demonstrated that MMP involvement in several diseases can be due to the hydrolysis of substrates other than ECM components, in particular chemokines involved in inflammation and immune response [1,12]. Ever since the first ligands had been disclosed almost 30 years ago, several MMP inhibitors (MMPIs) have been discovered, but none reached the market because of relevant side effects emerging in long-term treatment (musculoskeletal syndrome (MSS)) [13]. Traditional MMPIs are constituted by a functional group binding the zinc ion (zinc binding group (ZBG)) and chemical functions mimicking the substrate and interacting with the MMP subsites, in particular with the hydrophobic S1’ site. The ZBG is represented in most of the cases by the hydroxamate function, that binds very efficiently in the MMP active site, but can possibly bind other divalent cations and cause an unselective inhibition of other metallo-enzymes.

Another important issue of MMP inhibition is represented by the unselective inhibition of the studied MMPIs. In fact, clinically-tested compounds (e.g., batimastat and marimastat) were unselective MMP inhibitors not discriminating among different isoforms; while, during the years, the diverse role played by each MMP has been better understood [14]. In particular, in anticancer therapy, it has been defined that some MMPs can be considered therapeutic targets (MMP-1, MMP-2, MMP-7), while some others are classified as anti-targets (MMP-8) [15].

These issues prompted the search for alternative approaches to the MMP inhibition [16], such as the substitution of the ZBG with less potent, more selective zinc binders [17,18,19,20,21,22], the use of MMP-directed antibody [23] and the design of non-zinc binding inhibitors (NZIs) [24]. These ligands, commonly reported as third-generation MMPIs, have been developed for MMP-13 [25], MMP-8 [26] and MMP-12 [27] and can reach a better selectivity by missing the ZBG. Recently, we attempted to identify MMP-2 NZIs characterized by enhanced interactions with the S1’ pocket [28]. Our interest toward MMP-2 selective inhibition is due to the demonstrated role of MMP-2 in tumors. MMP-2, also called gelatinase A, is constitutively expressed and contributes to tissue homeostasis. Even if poorly inducible, MMP-2 has been found overexpressed in several tumors playing a role in both tumorigenesis and cancer progression [29].

MMP-8 and MMP-13 are classified as collagenases because they mainly degrade collagen; MMP-13 is able to degrade the components of the basal membrane and is involved in tumor dissemination and progression. On the contrary, MMP-8 has a confirmed protective role in tumor progression, and its inhibition should be avoided [29]. The final aim of our study is, therefore, to identify selective NZIs blocking MMP-2 and MMP-13 activity, while sparing the antitarget MMP-8.

**Figure 1:**
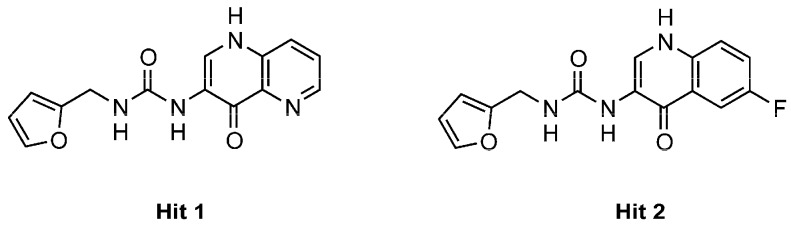
Chemical structures of **Hits 1** and **2**.

In the previous work, we moved from the analysis of the superposition between MMP-2 and MMP-8 in complex with a non-zinc-binding ligand that highlighted the possibility to design inhibitors tailored to (potential) additional interactions in the MMP-2 S1’ site. The following virtual screening campaign afforded one hit, the hydroxynaphtyridine derivative **Hit 1**, binding MMP-2 at a micromolar concentration (IC_50_ = 5 µM (MMP-2), 75 µM (MMP-8) and 36 µM (MMP-13)). An analogue testing allowed identifying also hydroxyquinoline compounds (i.e., **Hit 2**, IC_50_ = 15 µM (MMP-2), 105 µM (MMP-8) and 5 µM (MMP-13)), almost equally active than the prior hit (Figure 1).

Moving from these data, in the present work we investigated the structural determinants essential for the inhibition of MMP-2 by non-zinc-binding ligands. Activity data and docking studies of hydroxynaphtyridine/hydroxyquinoline derivatives suggested the role of relevant structural features:

- the furyl/aryl group that provides the π–π stacking with His201 (MMP-2 numbering);
- the CH_2_ spacer between the furyl/aryl group and the urea function;
- the urea function that provides H-bonds in the S1’ site with NH of Thr227 and CO of Leu218.

**Figure 2:**
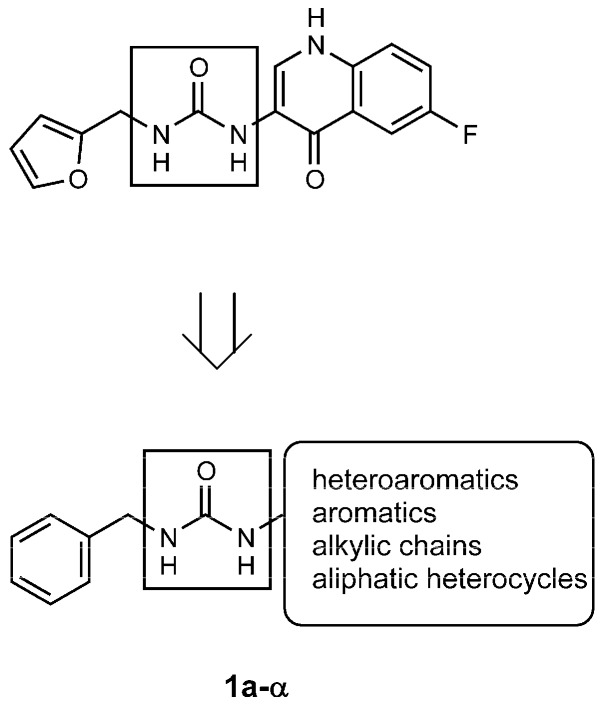
Structural modifications performed on **Hit 2**.

Starting from these evidences, we described the synthesis and the biological evaluation of new MMP inhibitors, based on the combination of three structural portions in which the lead compound could be ideally divided, in order to verify their importance for the selective inhibition of MMP isoforms. In all compounds, the furyl group was replaced by a more common benzyl function. In a first series of compounds, we kept the benzyl-ureidic scaffold unaltered, by modifying the hydroxyquinoline portion with heterocycles (quinoline, isoquinoline, indole, indazole), aromatic rings (naphthalene, benzene, pyridine), alkyl chains or saturated heterocycles (piperidine, piperazine, pyrrolidine) (Figure 2; Table 1).

**Figure 3:**
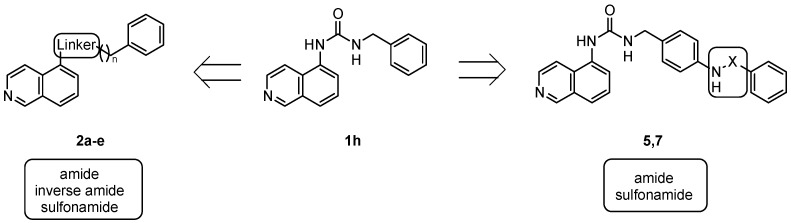
From isoquinolinyl urea **1h** to novel derivatives **2a**–**e** and **5**, **7**.

A second group of derivatives was obtained starting from the isoquinolinyl derivative **1h**, by replacing the ureidic linker with an amide or sulfonamide group (**2a**–**e**; Figure 3; Table 2). Finally, novel ureidic derivatives were designed by adding *para* substituents on the benzyl ring, with the aim to explore the effect of the polar functional group, potentially able to interact with residues at the top of the S1’ site (**5** and **7**; Figure 3; Table 3).

To provide some rational explanation of the activity data, structure-based computational approaches were applied to the study of the binding process.

## 2. Results

The present work moves from a previous study that identified MMP-2 non-zinc-binding hits (NZIs) [28]. As already stated, in this study, structural modifications were applied to these NZIs to probe the structural features determining the binding at and the selective inhibition of MMP-2, one of the most relevant MMPs in anticancer therapy. In this line, the final goal of our research project was the identification of NZIs particularly suitable for anticancer therapy, being selective toward target MMPs (such as MMP-2 and MMP-13) while sparing anti-target MMPs (such as MMP-8).

### 2.1. Chemistry

**Scheme 1:**
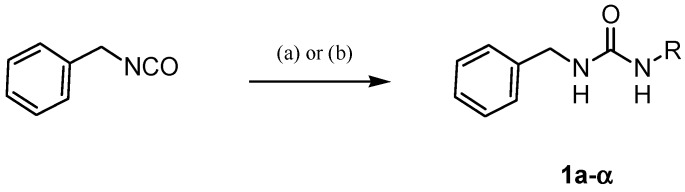
Reagents and conditions: benzyl isocyanate (1 equnivalent (eq)), amine, or aniline (1 eq), MeOH (Method a), or CHCl_3_, or dichloromethane (DCM) (Method b), room temperature (r.t.) or refluxing, 1–24 h, 41%–99%.

Urea derivatives **1a**–**α** (Table 1) were prepared by adding benzyl isocyanate to the corresponding amino derivatives in methanol, or chloroform, or dichloromethane, stirring the reaction at room or refluxing temperature for 1–24 h [30,31]. The synthesis is depicted in Scheme 1.

**Scheme 2:**
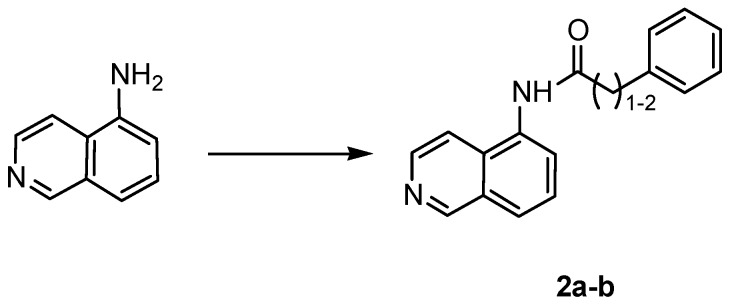
Reagents and conditions: 5-aminoisoquinoline (1 eq), phenylalkyl-chloride (1 eq), TEA (1.5 eq), dry DCM, N_2_, r.t., 2–24 h, 55%–63%; *n* = 1, **2a**; *n* = 2, **2b**.

**Scheme 3:**
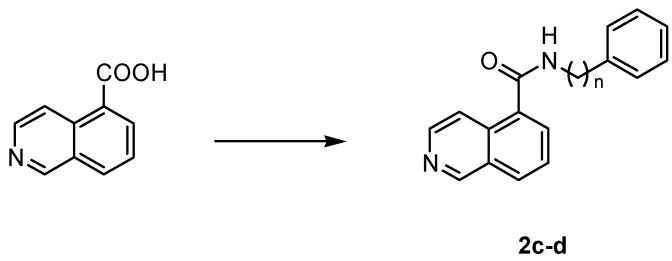
Reagents and conditions: 5-carboxyisoquinoline (1 eq), amine (1 eq), 1-hydroxybenzotriazole (HOBt) (1 eq), *N*,*N*-dicyclohexylcarbodiimide (DCC) (1 eq), *N*-methylmorpholine (NMM) (1 eq), DMF, 0 °C, r.t., 24 h, 58%–70%.

The synthesis of amides **2a**,**b** (Table 2) was carried out by adding to a solution of 5-aminoisoquinoline and triethylamine (TEA), a solution of suitable phenylalkyl-chloride in dichloromethane at room temperature, while stirring at r.t. for 2–24 h (Scheme 2) [32]. Amides **2c**,**d** were obtained by coupling 5-carboxyisoquinoline with benzylamine (for **2c**) or 2-phenylethylamine (for **2d**), in the presence of 1-hydroxybenzotriazole (HOBt), *N*,*N*-dicyclohexylcarbodiimide (DCC) and *N*-methylmorpholine (NMM), in DMF at 0 °C (Scheme 3).

**Scheme 4:**
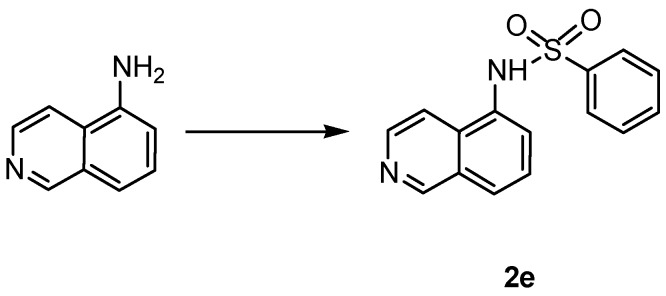
Reagents and conditions: 5-aminoisoquinoline (1 eq), phenylsulfonyl chloride (1.5 eq), pyridine, 0 °C, 3 h, 61%.

The sulfonamide **2e** was easily obtained by the reaction of 5-aminoisoquinoline with phenylsulfonyl chloride in pyridine at room temperature according to the literature (Scheme 4) [33,34].

**Scheme 5:**
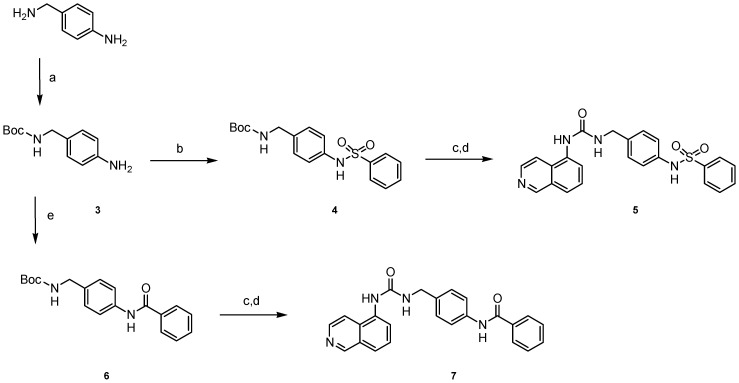
Reagents and conditions: (**a**) 4-aminobenzylamine (1 eq), di-tert-butyl-dicarbonate (Boc_2_O) (0.8 eq), THF, r.t., 2 h, 66%; (**b**) phenylsulfonyl chloride (1.2 eq), pyridine, 0 °C, r.t., 24 h, 86%; (**c**) 4 N HCl, dioxane, r.t., 4 h; (**d**) (i) TEA (1 eq), triphosgene (0.5 eq), toluene, reflux, 4 h; (ii) 5-aminoisoquinoline (1 eq), DCM, 0 °C, r.t., 18 h, 45%–48%; (**e**) benzoyl chloride (1.2 eq), pyridine, 0 °C, r.t., 24 h, 72%.

The synthetic route to derivatives **5** and **7** (Table 3) is illustrated in Scheme 5. The 4-aminobenzylamine was monoprotected by tert-butyloxycarbonyl (Boc) in THF, at room temperature; the intermediate **3** was then reacted with phenylsulfonyl chloride, in pyridine at 0 °C. The resulting sulfonamide **4** was deprotected in acidic conditions, followed by a treatment with triphosgene and 5-aminoisoquinoline, in the presence of TEA. In this step, the intermediate isocyanate, formed by treatment with triphosgene, was not isolated, but it was reacted in situ to obtain the desired urea **5**.

The amide derivative **7** was obtained by starting from intermediate **3**, with the same synthetic procedure.

### 2.2. Biochemical Assays

**Figure 4:**
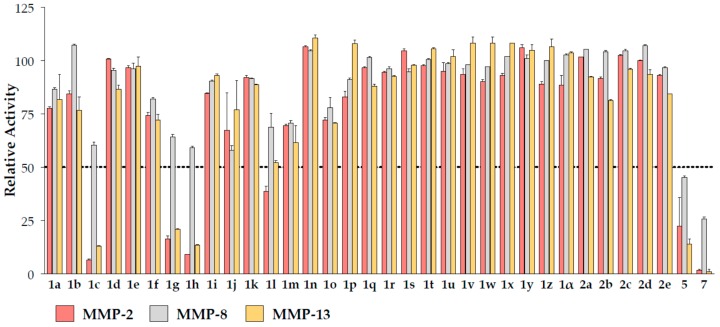
Residual enzyme activity on MMP-2, MMP-8 and MMP-13 of Compounds **1a**–**α**, **2a**–**e**, **5** and **7** at 100 µM.

The synthesized compounds were submitted to MMP inhibition assays. A dose/response preliminary screening was performed at 100 µM by registering the residual enzyme activity on MMP-2, MMP-8 and MMP-13 (Figure 4). Compounds showing a percentage of inhibition higher than 50% were submitted to IC_50_ calculation. Activity data are reported in Table 4.

The **1h** resulted the most active compound among the first two series of compounds (**1a**–**α**, **2a**–**e**), while tight analogues, such as **1i**, were inactive. The active compounds showed an interesting selectivity with respect to MMP-8.

The inhibition assays allowed to evidencing in detail the role played by specific structural features characterizing the set of NZIs: (i) the hydroxyl group on the quinoline ring (**Hit 2**) does not lead to detectable improvement of MMP inhibition; (ii) either urea → amide simplification or decreasing the bicycle hindrance led to inactive or less active compounds; (iii) the presence of nitrogen on the bicyclic ring is a requirement for activity, while only definite positions in the quinoline moiety led to active ligands.

In the attempt to improve inhibition activity, we extended the molecular structure of these inhibitors, to tentatively approach the residues on the top of the S1’ site, which are usually involved in binding with H-bond acceptor groups (generally the sulfonamide oxygen atoms) [35]. To this aim, *p*-phenylsulfonylamide or *p*-phenylamide were introduced on the benzyl group to yield Compounds **5** and **7**, respectively (Table 4). These compounds proved to be active toward all of the assayed MMPs. Compared to **1h**, Compound **7** presented an improved inhibition potency toward MMP-2 and MMP-13, although with a reduced selectivity toward MMP-8.

In order to elucidate the inhibition assay outcomes based on the structure of MMP-NZI complexes, computational studies were carried out by using either docking or molecular dynamics procedures.

### 2.3. Docking

**Figure 5:**
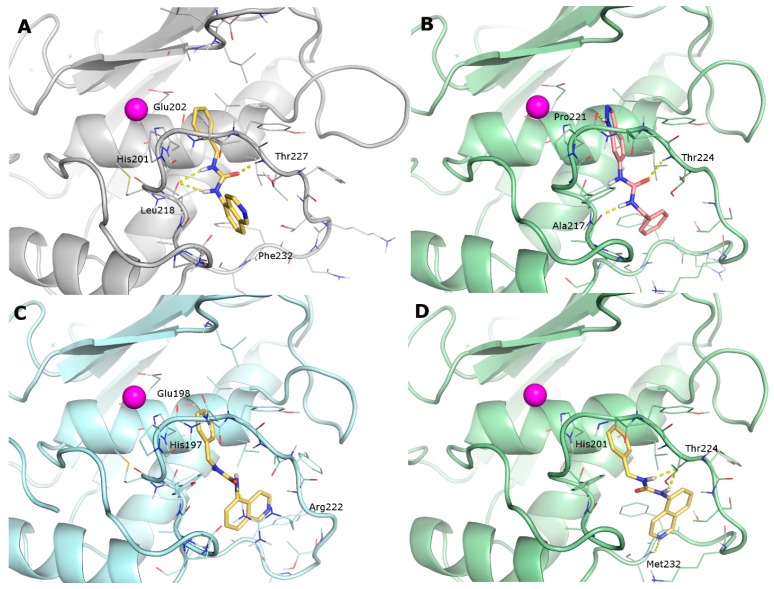
Docking poses of: (**A**) **1h** in the MMP-2; (**B**) **1m** in the MMP-13; (**C**) **1h** in the MMP-8; and (**D**) **1h** in the MMP-13. MMPs are represented as grey (MMP-2), pale cyan (MMP-8) and pale green (MMP-13) cartoons. The zinc ion is a purple sphere; most relevant residues are represented as lines and ligands as sticks. H-bonds are depicted as yellow dashed lines.

Docking calculations of the synthesized ligands in the MMP-2, MMP-8 and MMP-13 catalytic site were performed using Glide. Receptor structures were selected through a cross-docking study (Supplementary Materials), and docking calculations were conducted at both standard precision (SP) and extra precision (XP) settings, with and without a water molecule on the zinc ion. Above all, the binding mode of the synthesized ligands is similar to the one described for the **Hits 1** and **2**. As an example, the docked pose of **1h** in the MMP-2 binding site showed that the benzyl ring stacks on the His201 imidazole ring; the urea NHs form H-bonds with Leu218 CO; while the carbonyl oxygen is connected to the Thr227 NH. The isoquinoline ring is mainly solvent-exposed and only partially buried by hydrophobic residues in the lower part of the S1’ site (Leu218, Phe232) (Figure 5A). A similar pattern of interactions was found in all MMP-2-NZI docking complexes, including both active and inactive ligands. The binding of indazole derivatives (**1l** and **1m**) is inverted in all receptors with the heterocycle NH H-bonded to Pro221 CO (Figure 5B).

In the MMP-8 active site, the presence of the Arg222 side chain hinders the S1’ site and limits the positioning of the bulky isoquinoline ring, giving a reason for the observed selectivity (Figure 5C).

On the contrary, the larger specificity site of MMP-13 allows easily accommodating the studied ligands in a binding mode similar to that of well-known NZIs [25]: the benzyl ring forms a π–π stacking with His201; the urea NHs are engaged in H-bonding with Thr224 CO; while the isoquinoline N forms a H-bond with the Met232 NH (Figure 5D).

**Figure 6:**
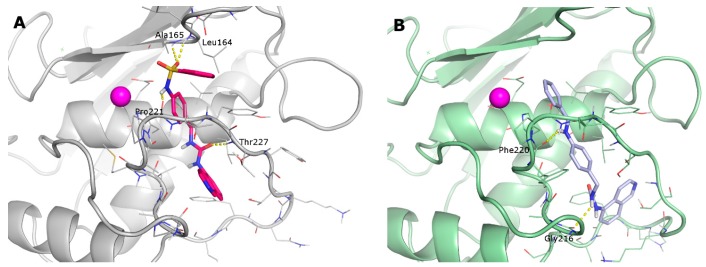
Docking poses of: (**A**) ligand **5** in the MMP-2; (**B**) and ligand **7** in the MMP-13. MMPs are represented as grey (MMP-2), and pale green (MMP-13) cartoons. The zinc ion is a purple sphere; most relevant residues are represented as lines and ligands as sticks. H-bonds are depicted as yellow dashed lined.

Additional interactions were evidenced by the docking of larger ligands (**5** and **7**): in MMP-2, these compounds interact with Leu164 and Ala165 NH, while in MMP-13, they occupy the S1’ site with a banana-shape, typical of MMP-13 NZIs (Figure 6).

**Figure 7:**
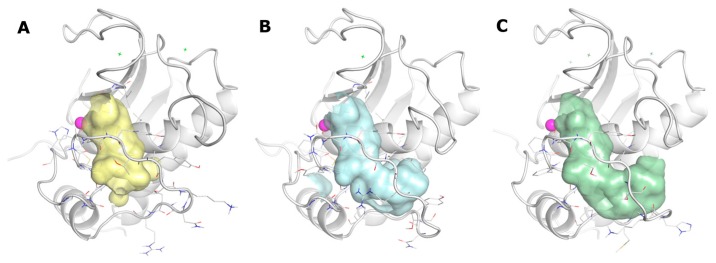
The molecular surface of the S1’ site of, (**A**) MMP-2 (pale yellow), (**B**) MMP-8 (pale cyan) and (**C**) MMP-13 (pale green) is depicted. MMP-2 has a tunnel-like S1’ site, while the MMP-8 and MMP-13 S1’ loop moves, opening an accessory pocket called the S1’* site. However, the MMP-13 site is much larger, the MMP-8 site being hindered by the Arg222 side chain.

To have a better comprehension of the described SAR, the surface of the S1’ site of the studied MMPs has been represented, showing the different shapes of the three hydrophobic pockets (Figure 7).

From a quantitative point of view, no correlation between the experimental activity and scoring values can be provided by docking calculations. Moreover, the different potency observed for close analogues, i.e., the **1h** and **1i** ligands, cannot be easily explained based on their similar binding mode. In the latter example, it is not clear the role played by the nitrogen atom in the inhibition process, because it seems to not appreciably interact with the enzyme in the docking models of either **1h** or **1i** bound complexes. Thus, molecular dynamics (MD) simulations were carried out to include the effects of induced fit and the interaction with explicit water molecules into the binding model. In particular, we focused on MMP-2, as it is an important therapeutic target, and just a few NZIs have been identified so far [28,36,37]. The binding of the above-mentioned **1h** and **1i** ligands was studied, being representative of the case of highly similar ligands characterized by highly different inhibition activities.

### 2.4. Molecular Dynamics

**Figure 8:**
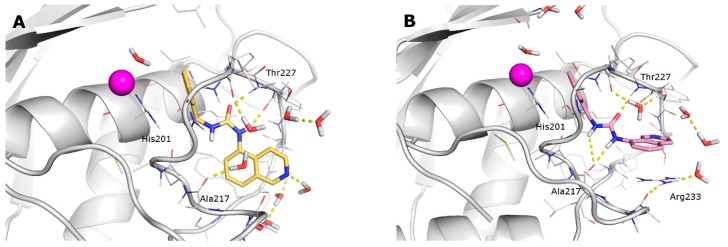
Binding poses of **1h** (**A**) and **1i** (**B**) obtained from the last 90 ns of the MD trajectory. MMP-2 is represented as a grey cartoon and ligands as sticks. H-bonds are depicted as yellow dashed lines.

The docking complexes of **1h** and **1i** were subjected to molecular dynamics simulations as reported in the Materials and Methods section. In spite of the high similarity in the ligand structures and starting poses, the **1h** and **1i** bound complexes required about 100 and 10 ns, respectively, to gain trajectory stabilization (Figure S1). On the one hand, the starting (obtained by docking) pose of **1i** was essentially maintained and, thus, induced a softer adaptation of the MMP-2 conformation. The **1i** binding pose observed in the stabilized trajectory (last 90 ns) is characterized by essentially the same interactions with MMP-2 detected in the corresponding docking pose (Figure 8B), although the presence of explicit waters allowed improving the description of the **1i** binding domain.

On the other hand, we detected a marked rearrangement of the **1h** conformation consisting of the *trans* → *cis* conversion of the amide bond between the ureidic C=O and the 5-aminoisoquinoline (Figure 8A). This significant conformational change is responsible for the longer simulation time required for the MMP-2 adaptation to the new ligand pose (Figure S1).

To corroborate the occurrence of amide bond rearrangement in the binding of **1h** at MMP-2, density functional theory (DFT) calculations were performed to investigate the thermodynamics and kinetics of this isomerization process. Calculations indicated that, as expected, the *trans* is more stable than the *cis* conformation by 0.84 kcal/mol (0.38 kcal/mol in the gas phase), corresponding to a *trans*/*cis* ratio of 96:4. Thus, although the *trans* conformation is predominant, the amount of *cis* conformer is not negligible at equilibrium. Therefore, we estimated a very low kinetic barrier of 7.51 kcal/mol (7.50 kcal/mol in the gas phase), indicating that *cis*/*trans* equilibrium is rapidly gained. Above all, calculations evidenced that the formation of a MMP-2:**1h** complex with the ligand in the *cis* conformation cannot be excluded or that the *trans* → *cis* interconversion may feature the binding pose of these ligands that would be represented by time-averaged structures having the *cis* and *trans* conformations as limiting terms.

MD calculations provided for additional information on the bound structure of MMP-2 and on the occurrence of *trans* → *cis* amide rearrangement potentially affecting the binding of these inhibitors. Thus, the stable trajectories of **1h** and **1i** complexes were processed to extract representative bound conformations of MMP-2 by means of a clustering analysis tool. The target conformations obtained from clustering are shaped by the binding of ligands in either *trans* (**1i**) or *cis* (**1h**) conformation and by the interaction with explicit water molecules. The two plus two protein conformations gained from the clustering of the two bound complexes show that MMP-2 maintains a global similarity in the shape of the ligand binding pocket, warranting the π–π stacking of the benzyl ring at His201 and the same number of hydrogen bond contacts of the ureidic moiety for both **1h** and **1i** ligands, with the main rearrangement involving the Arg233 side chain that, in the two complexes with **1i**, bends toward the ligand to provide a cation–π interaction. Another difference evidenced in the representative structures of bound complexes was found in the number of hydrogen bond contacts being 138 and 129 for the MMP-2:**1h** complex structures and 135 and 124 for the MMP-2:**1i** complex, highlighting that also non-local effects may contribute to differentiating the affinity of these ligands for MMP-2.

A newly-directed docking study was then performed by applying two changes to the standard protocol based on the MD results: (i) MMP-2 receptor structures obtained through the clustering of both **1h** and **1i** MD trajectories were assembled to have a multi-conformational model of the target (ensemble docking), hence encoding a higher adaptation to the synthesized compounds, either active or inactive; (ii) ligand conformations affected by the *trans* → *cis* rearrangement of the ureidic moiety were assigned with no energy penalty, i.e., increasing the probability of harvesting docking poses in the *cis* conformation. Ensemble docking calculations were thus carried out by using the four-conformational model of MMP-2 and by probing all studied ligands in each receptor conformation to finally assign one pose per ligand with the optimal scoring and interaction mapping. The binding mode of **1h** and **1i** ligands resulting from the ensemble docking calculations are similar to the ones obtained in the first docking campaign; no *trans* conformation was in fact detected among the best poses of ensemble docking. The ensemble docking procedure was not able to provide evidence for the binding of the ligand in the *cis* conformation.

**Figure 9:**
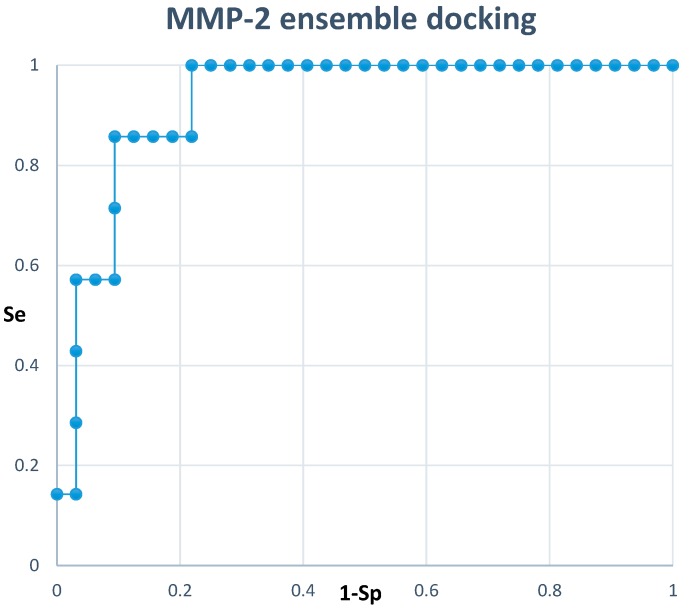
ROC plots obtained reporting the ranking of active compounds after ensemble docking.

On the other hand, the use of ensemble docking allowed enhancing the correlation between the activity and the score and, more specifically, to gain a higher enrichment, as indicated by the ROC curve reporting the ability of our model to provide a higher score for more active compounds (AUC = 0.91) (Figure 9).

## 3. Discussion

The objective of the present study was the identification of new NZIs selective toward MMP-2 and MMP-13, which are involved in cancer development. The approach we followed was aimed to overcome two main issues connected to MMP inhibition: (i) the presence of a chemical function binding the catalytic zinc ion, which could be responsible, in several cases, for some side effects emerging in clinical trials, as already discussed above; (ii) the need for isoform selective ligands, because each MMP plays a different role in possibly pathological conditions, as underlined recently [6].

While MMP-13 NZIs have been widely investigated [25], there are only a few examples of MMP-2 non-zinc-binding ligands [28,36,37]; therefore, our focus was to depict the binding process of newly-designed inhibitors, gaining more insight into the essential structural features for MMP-2 non-zinc binding inhibition, in order to drive the design of the selective and most potent ligands.

The structural determinants of isoform-selective NZIs were investigated through the synthesis and the biological evaluation of 34 newly-designed ligands tested against MMP-2, MMP-8 and MMP-13. In the previous study [28], a virtual screening campaign identified new inhibitors able to block the MMP-2 activity not containing the ZBG. Docking studies suggested the role of the aryl group facing His201, such as the necessary CH_2_ spacer between this aromatic group and the urea. In this work, by simplifying the **Hit 2** structure, we confirmed previous observations about the role of the urea function, which is that simplification did not enhance the inhibition activity. Moreover, we verified that the hydroxyl group of **Hit 2** is not necessary for inhibition, but the bicyclic hindrance must be maintained, and the nitrogen atom on a good definite position of the quinoline moiety is required to ensure inhibition.

More sized inhibitors, including *p*-phenylsulfonylamide or *p*-phenylamide substitution of the benzyl ring, i.e., Compounds **5** and **7**, respectively, were also synthesized to promote interactions with residues on the top of the S1’ site, as this interaction is frequently observed in traditional MMPIs. Results demonstrated that our objective has been possibly reached because these compounds were both active toward all of the assayed MMPs, and more specifically, Compound **7** resulted in being a more potent inhibitor of MMP-2 and MMP-13 compared to **1h**, although with a reduced selectivity toward MMP-8. This result can be considered as a confirmation of the binding mode of these compounds that has been suggested by docking calculations.

Computational studies were then carried out by the use of either docking or molecular dynamics procedures to elucidate the inhibition assay outcomes and shed some light on the structure-activity relationships affecting the set of investigated NZIs.

Docking calculations provided a sketch of the binding pose at MMP-2, MMP-8 and MMP-13 for the synthesized ligands. All compounds, either active or inactive, shared a similar binding pose, showing the stacking of the benzyl ring on the His201 imidazole ring, the same donating and accepting H-bond contacts of urea with S1’ site residues and the solvent exposure of the bicyclic ring (see Docking subsection in the Results section).

However, no explanation of the activity data was provided by the docking results that assigned a similar score both to active and inactive ligands. These results evidenced that if, on the one hand, our docking approach provided for an insight into the target-ligand binding geometries, on the other hand, it may lead to an approximate estimation of the target-ligand affinities, probably because some significant aspects of the binding process are neglected or less accurately included in the model. Indeed, although reputed important determinants of the binding process, either receptor flexibility and adaptability to the ligands (induced fit effects) or explicit water molecule interactions are usually omitted in docking calculations.

Molecular dynamics (MD) simulations were then carried out to include these latter ones in the binding model of **1h** and **1i** at MMP-2. These calculations aimed mostly at providing an explanation for the drastic response on the MMP-2 inhibition activity of **1h** and **1i** caused by changing only the position of the nitrogen in the quinoline ring. From the stabilized trajectory of the MMP-2:**1i** complex, we detected essentially the same target-ligand interactions obtained from docking, although the presence of explicit waters may potentially influence the accommodation of the ligand.

An unexpected *trans* → *cis* rearrangement of the ligand conformation, affecting the amide bond connecting the ureidic moiety to the isoquinoline ring, resulted from the MD simulation of the MMP-2:**1h** complex. Density functional theory (DFT) calculations indicated that although the *trans* asset is more stable than the *cis* one, the *trans*/*cis* ratio can be estimated in 96:4 and, thus, determines a non-negligible amount of *cis* conformer at equilibrium. These calculations evidenced that the investigated NZIs could be subjected to a *trans* → *cis* interconversion in the binding of MMP-2 or, at least, that this hypothesis cannot be ruled out. This *cis*, less stable, conformation, therefore, can be stabilized by H-bonding interaction with water molecules, not observed for the **1i** ligand. This result suggests the role of water molecules in determining the activity of quinoline/isoquinoline derivatives depending on the position of the nitrogen atom. Further interesting data emerging from MD are the number of intramolecular H-bonds of the complex: complexes with ligand **1h** have a higher number of H-bonds accounting for a stabilization of the whole system. A similar result has been observed in a previous study that applied docking and molecular dynamics to an analogue case-study on MMP-2 [38]. Furthermore, in the present study, we can confirm that the MMP-2 inhibition process cannot be studied by applying simple docking calculations.

The stable trajectories of **1h** and **1i** complexes were clustered to extract representative bound conformations of MMP-2, and a newly-directed, ensemble docking study was then performed. The ensemble docking procedure was not able to provide evidence for the binding of ligand in *cis* conformation, possibly for two reasons: (i) the *trans* → *cis* interconversion may be an intrinsic feature of a ligand bound at MMP-2, so that a single representative pose would be an average of a *cis*/*trans* bundle of structures; docking poses are instead calculated as minimum structures, thus making it rather unlikely to obtain a binding pose intermediate between *cis* and trans; (ii) explicit water molecules, which are automatically removed by the standard receptor preparation tool (see the Materials and Methods), might be essential to prompt the ligand into a *cis* or *trans* conformation, and their exclusion from the binding model may turn out to be a too marked approximation. On the other hand, the ensemble docking allowed enhancing the correlation between activity and score, gaining a higher enrichment in the ROC curve and enabling the application of this model to study the binding of new derivatives.

## 4. Materials and Methods

### 4.1. Chemistry

#### 4.1.1. General

Melting points were determined with a Büchi Melting Point B-450 (Büchi Labortechnik, Flawil, Switzerland). NMR spectra were recorded on a Varian Mercury 300 spectrometer (Varian, Palo Alto, CA, USA) with ^1^H at 300.060 MHz and ^13^C at 75.475 MHz. Proton chemical shifts were referenced to the tetramethylsilane (TMS) internal standard. Chemical shifts are reported in parts per million (ppm, δ units). Coupling constants are reported in Hertz (Hz). Splitting patterns are designed as: s, singlet; d, doublet; t, triplet; q, quartet; ql, quartet like; dd, double doublet; m, multiplet; b, broad.

All commercial chemicals and solvents are reagent grade and were used without further purification unless otherwise specified. Reactions were monitored by thin-layer chromatography on silica gel plates (60F-254, E. Merck, Merck Group, Darmstadt, Germany) and visualized with UV light, iodine vapors or alkaline KMnO_4_ aqueous solution. The following solvents and reagents have been abbreviated: triethylamine (TEA), tetrahydrofuran (THF), dimethyl sulfoxide (DMSO), ethyl acetate (EtOAc), dichloromethane (DCM), dimethyl formamide (DMF), methanol (MeOH), ethanol (EtOH), chloroform (CHCl_3_). All reactions were carried out with the use of standard techniques.

#### 4.1.2. General Procedure for the Synthesis of Ureas **1a**–**α**

Benzyl isocyanate (1 eq) was added to a solution of the suitable amine or aniline (1 eq) in 10 mL of MeOH (Method a), or CHCl_3_, or DCM (Method b), and the mixture was allowed to react at r.t. or refluxing temperature from 1–24 h. The solvent was removed under reduced pressure, and crude products were purified by crystallization or column chromatography.

#### 4.1.3. General Procedure for the Synthesis of Amides **2a**,**b**

To a solution of 5-aminoisoquinoline (1 eq) and TEA (1.05 eq) in DCM (3.0 mL) at room temperature was added a solution of phenylacetyl chloride (Compound **2a**, 1.05 eq) or 3-phenylpropanoyl chloride (Compound **2b**, 1.05 eq), respectively. The mixture was allowed to react at r.t. for 2–24 h. After this time, the solvent was removed under reduced pressure; the crude material was treated with saturated NaHCO_3_ (20 mL) and extracted with DCM (3 × 20 mL). The organic phase was washed with brine (2 × 60 mL), dried on sodium sulfate and evaporated under reduced pressure. Crude products were purified by crystallization.

#### 4.1.4. General Procedure for the Synthesis of Amides **2c**,**d**

1-Hydroxybenzotriazole (HOBt) (1 eq) and *N*,*N*-dicyclohexylcarbodiimide (DCC) (1 eq) were added to a solution of 5-carboxyisoquinoline (1 eq) in DMF (4 mL) at 0 °C. After 10 min, *N-*methylmorpholine (1 eq) and the proper amine (1 eq) (benzylamine or phenylethylamine) were added, and the reaction was allowed to reach room temperature.

After stirring overnight, the solvent was removed under reduced pressure; the crude material was treated with saturated NaHCO_3_ (20 mL) and extracted with DCM (3 × 20 mL). The organic phase was washed with brine (2 × 60 mL), dried on sodium sulfate and evaporated under reduced pressure. Crude products were purified by column chromatography.

#### 4.1.5. General Procedure for the Synthesis of Carbamates **4** and **6**

To a solution of **3** (1.2 g, 5.4 mmol) in pyridine (8 mL) was carefully added phenylsulfonyl chloride or benzoyl chloride (6.5 eq) at 0 °C, and the temperature was allowed to reach room temperature. After 24 h, the mixture was neutralized by 2 N HCl at 0 °C, then extracted with DCM (3 × 20 mL). The organic phase was dried on sodium sulfate and the solvent evaporated under reduced pressure. Crude materials were purified by column chromatography.

#### 4.1.6. General Procedure for the Synthesis of Ureas **5** and **7**

A solution of the suitable carbamate (**4** or **6**, 1 eq) in dioxane was treated with HCl 4 N (5 mL) and stirred at room temperature for 4 h. After removal of volatiles, the deprotected hydrochloric salt was added to a solution of triphosgene (0.5 eq) in toluene (10 mL), in the presence of TEA (1 eq), and the mixture was refluxed for 4 h. After evaporation of toluene under reduced pressure, the solid obtained was dissolved in DCM (10 mL) and treated with the 5-aminoisoquinoline (1 eq) at 0 °C. The reaction was stirred at room temperature overnight, then concentrated to dryness, diluted with EtOAc (15 mL) and washed with HCl 2N (3 × 15 mL) and brine (3 × 15 mL). The organic solution was dried over sodium sulfate and evaporated under reduced pressure. Crude materials were purified by crystallization.

### 4.2. Enzyme Inhibition Assay

The catalytic domains of MMP-2, MMP-8 and MMP-13 were purchased from Enzo Life Sciences (Lörrach, Germany). The assays were performed in triplicate in 96-well white microtiter plates (Corning, NBS; Corning, NY, USA). For assay measurements, inhibitor stock solutions (DMSO, 10 mM) were diluted to six different concentrations (1 nM–100 µM) in fluorometric assay buffer (50 mM Tris·HCl pH 7.5, 200 mM NaCl, 1 mM CaCl_2_, 1 µM ZnCl_2_, 0.05% Brij-35 and 1% DMSO). Enzyme and inhibitor solutions were incubated in the assay buffer for 15 min at room temperature before the addition of the fluorogenic substrate solution (OmniMMP^®^ = Mca-Pro-Leu-Gly-Leu-Dpa-Ala-Arg-NH_2_, Enzo Life Sciences, 2.5 µM final concentration or OmniMMP^®^RED = TQ3-GABA-Pro-Cha-Abu-Smc-His-Ala-Dab(6’-TAMRA)-Ala-Lys-NH_2_, (TAMRA: tetramethylrhodamine) Enzo Life Sciences, 1 µM final concentration). After further incubation for 2–4 h at 37 °C, fluorescence was measured (λ_ex_ = 340 nm, λ_em_ = 405 nm or λ_ex_ = 545 nm, λ_em_ = 572 nm) using a Perkin-Elmer Victor V3 plate reader (Waltham, MA, USA). Control wells lacked inhibitor. The MMP inhibition activity was expressed in relative fluorescence units (RFU). Percent inhibition was calculated from control reactions without inhibitor; IC_50_ values were determined using GraphPad Prism version 5.0f and are expressed as the mean ± SEM of at least three independent measurements in triplicate.

### 4.3. Computational Methods

#### 4.3.1. Ligand Preparation

The structures of synthesized ligands were manually built exploiting the Build facility in Maestro version 10.2 [39]. The protonation state at pH 7.4 and possible tautomers were generated using the Ligand preparation routine in Maestroand applying the following parameters: force field OPLS2005, generation of the protonation state using Epik version 3.2. The obtained structures were minimized to a derivative convergence of 0.01 kJ·Å^−1^·mol^−1^, using the Polak–Ribiere conjugate gradient (PRCG) minimization algorithm, the OPLS2005 force field and the eneralized Born/Surface Area (GB/SA) water solvation model implemented in MacroModel version 10.8 [39]. The conformational search was carried out on all minimized ligand structures, applying the mixed-torsional/low-mode sampling, the OPLS2005 force field, the water solvation model GB/SA and the automatic setup protocol, in order to obtain the global minimum geometry of each molecule, which was then submitted to docking calculations.

#### 4.3.2. Docking

3D structures of MMP-2, MMP-8 and MMP-13 were retrieved from the Protein Data Bank repository [40]. Only complexes with resolution ≤2.5 Å were selected (Supplementary Material, Table S1). Each MMP structure was submitted to the Protein Preparation wizard in Maestro [39] that allows eliminating ligands and water molecules, fix bond orders, add hydrogen atoms, compute the residue protonation state, optimize the H-bond network and relax the structure with a constrained minimization. All structures were superimposed to have the same frame of reference. The Glide grid generation protocol (Glide version 3.9 [39]) was applied to each structure using the same coordinates for the grid box positioning (*x* = 67.1815; *y* = 25.4097; *z* = 27.9457). The so-prepared structures were submitted to a preliminary cross-docking study (cross-docking details and results are reported in the Supplementary Material, Tables S2–S4) that allowed the selection of the best performing 3D structures, used for the following docking calculations. PDB IDs of selected 3D structures are: 3AYU and 1QIB for MMP-2, 1KBC and 3DPF for MMP-8; and 2OZR for MMP-13.

The global minimum conformations of the synthesized ligands were used as the input geometries for docking calculations in the previously-mentioned MMP catalytic sites. The docking calculations were carried out using the Virtual Screening Workflow. Both Glide SP (standard precision) and XP (extra precision) methods were used applying the default parameters [39]. Ten poses were saved for each ligand. The same protocol (protein preparation, grid generation and docking) was applied to the MMP-2 structures obtained from MD trajectory clustering. A total of four MMP-2 structures (corresponding to the two clusters for each complex; see below) were used for the final docking runs.

#### 4.3.3. Molecular Dynamics

The docking structures of either or MMP2:**1h** or **1i** complexes used as starting models in subsequent molecular dynamics (MD) simulations with the Gromacs package [41]. Each complex was placed in a cubic box whose dimensions prevent self-interaction and then solvated with up to 9893 water molecules, at the typical density of water (298 K, 1.0 atm) and by employing the 4-site transferable intermolecular potential function (TIP4) water model [42,43]. The molecular systems were simulated in the Amber99 force field [44]. Sodium and chloride ions were added as counterions, to ensure electrical neutrality. Simulations were initially performed by the following scheme:

(i) Local energy minimization, heating up to 300 K in six MD runs (100 ps) with protein temperature set at 0, 100, 150, 200, 250 and 300 K;
(ii) Production runs of 100 ns at 300 K in an isothermal–isobaric (NPT) ensemble, by using the velocity rescaling scheme (temperature) and the isotropic Berendsen coupling scheme (pressure) [45];
(iii) The bound proteins were simulated in the Amber force field [46,47]; the linear constrain solver (LINCS) algorithm was adopted to constrain all bond lengths [48], and the long range electrostatics were computed by the particle mesh Ewald method [49].

Such a procedure provided 90 ns of stable trajectory only for the MMP-2:**1i** complex, whereas longer equilibration times were required by MMP-2:**1h** simulation, thus corresponding to a different scheme:

(i) Local energy minimization, heating up to 300 K in six runs with protein temperature set at 0 (100 ps), 100 (200 ps), 150 (200 ps), 200 (200 ps), 250 (200 ps) and 300 K (200 ps);
(ii) Production runs of 200 ns at 300 K in an NPT ensemble, by using the velocity rescaling scheme (temperature) and the isotropic Berendsen coupling scheme (pressure);
(iii) Same setting of MMP-2:**1i** simulation.

Bonded and Van der Waals parameters for the description of the Zn^2+^ ions’ first coordination sphere were retrieved from [50], whereas atomic charges were calculated at the density functional theory (DFT) level of theory by using Gaussian 09 [51] (See below). The Amber parameters for the description of ligand **1i** structures were mostly implemented in the Gromacs parameter file, and only atomic charges were calculated quantum mechanically with Gaussian 09 (See below).

Trajectory analyses were carried out by using the Gromacs utilities, VMD [52] and Maestro [39] graphical interfaces. Ensembles of 900 snapshots per trajectory (one for 100 ps) were extracted, analyzed and clusterized by using the *g*_cluster utility. Clustering was employed to sample representative configurations of the bound proteins to be used in subsequent docking calculations. Either MMP-2:**1h** or MMP-2:**1i** trajectory ensembles were clusterized by using the method proposed by Daura et al. [53] and a cut-off of 1.5 Å by the comparison of the binding pose and the protein residues or water molecules within 3.0 Å of the ligand. The middle structures for the clusters covering at least 80% of the whole ensemble were eventually selected as representative.

#### 4.3.4. DFT Calculations

The local minimum geometries of free **1h** and **1i** in the gas phase were calculated at the B3LYP/6-31G* [54,55,56] level of theory by using Jaguar version 8.8 [39]. Atomic charges were then obtained by the fitting of the electrostatic potential by the restrained electrostatic potential (RESP) method [57] and subsequently employed in the MD simulation of the **1h** and **1i** bound complexes. The same computational scheme was also used for the calculation of the charges of the atoms within the first coordination sphere of the Zn^2+^ ions.

The investigation of the kinetics and thermodynamics of the cis/trans conversion of the **1h** ligand (vide infra) was performed by using the Gaussian 09 programs package [51]. The geometry of either the *cis* or *trans* conformation of **1h** was optimized in the gas phase at the B3LYP/6-31+G* [54,55,56] level of theory. A relaxed scan of the torsion around the ureidic-aminoisoquinoline amide bond was performed at the same level of theory to obtain a guess structure of the transition state for *cis*/*trans* conversion. The transition state structure was then obtained by optimizing the geometry with the maximum energy in the scan as a first-order saddle point by using the Berny algorithm [58,59] implemented in Gaussian 09. Vibrational frequency analyses were then performed at the same level of theory to confirm the correct nature of the optimized stationary points and to calculate the zero-point energy and thermal corrections (under the hypothesis of an ideal gas behavior) for free energy estimations. The integral equation formalism within the polarizable continuum model (IEFPCM) [60]) was used to correct the gas phase free energies of *trans*-**1h**, *cis*-**1h** and the corresponding trans-to-cis transition state structure (*ts*-**1h**) by the effect of water solvation.

## 5. Conclusions

In conclusion, with respect to previous work [28], in the present study, we obtained: (i) the synthesis of 34 new compounds with simplified structures; (ii) a better understanding of the SAR of these quinoline/isoquinoline derivatives, in particular concerning the role of the urea and quinoline ring; (iii) the validation of the hypothesized binding mode, suggesting the missing interaction with the zinc ion; (iv) a computational model based on the combination of docking and molecular dynamics procedures able to enhance the correlation between the activity and docking score in the computer-assisted design of new MMP-2 NZIs.

Above all, the combination of experimental and computational outcomes provided by the present investigation shed some lights on the structure-activity and structure-selectivity affecting the investigated compounds and paves the way to future efforts in the development of newly-designed NZIs.

## Figures and Tables

**Table 1:** Urea derivatives **1a**–**α**.

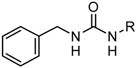			
**ID**	**R**	**ID**	**R**
**1a**	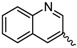	**1n**	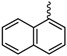
**1b**	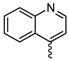	**1o**	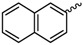
**1c**	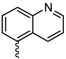	**1p**	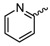
**1d**	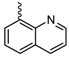	**1q**	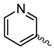
**1e**	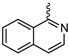	**1r**	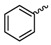
**1f**	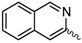	**1s**	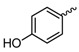
**1g**	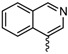	**1t**	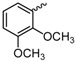
**1h**	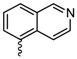	**1u**	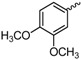
**1i**	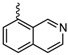	**1v**	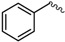
**1j**	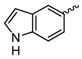	**1w**	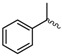
**1k**	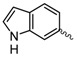	**1x**	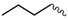
**1l**	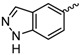	**1y**	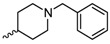
**1m**	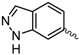	**1z**	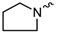
**1α**	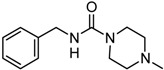		

**Table 2:** Isoquinoline amide and sulfonamide derivatives **2a**–**2e**.

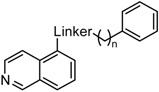		
**Cpd**	**Linker**	***n***
**2a**	NHCO	1
**2b**	NHCO	2
**2c**	CONH	1
**2d**	CONH	2
**2e**	NHSO_2_	-

**Table 3:** Amide and sulfonamide derivatives of isoquinolinyl urea **5** and **7**.

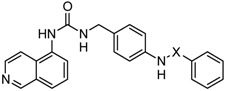	
**Cpd**	**X**
**5**	SO_2_
**7**	CO

**Table 4:** Activity data (IC_50_, µM) on MMP-2, MMP-8 and MMP-13.

ID	MMP-2	MMP-8	MMP-13
**1c**	55 ± 1	>100	58 ± 1
**1g**	74 ± 3	>100	73 ± 11
**1h**	15 ± 4	>100	14 ± 4
**1l**	86 ± 2	>100	98 ± 2
**5**	54 ± 7	88 ± 5	55 ± 7
**7**	7.4 ± 0.8	48.5 ± 1.6	6.6 ± 0.4

## Supplementary Materials

Supplementary materials can be found at www.mdpi.com/1422-0067/17/10/1768/s1.
