# Supplementary material for: Seeking for Non-Zinc-Binding MMP-2 Inhibitors: Synthesis, Biological Evaluation and Molecular Modelling Studies
Source: Int J Mol Sci. 2016 Oct 22;17(10):1768. doi: 10.3390/ijms17101768 (PMC5085792; doi:10.3390/ijms17101768)
Supplement: Supplementary file 1 [file ijms-17-01768-s001.pdf]

Supplementary Materials: Seeking for Non-Zinc-Binding MMP-2 Inhibitors: Synthesis, Biological Evaluation and Molecular Modelling Studies

Alessandra Ammazzalorso, Barbara De Filippis, Cristina Campestre, Antonio Laghezza, Alessandro Marrone, Rosa Amoroso, Paolo Tortorella and Mariangela Agamennone

Cross-docking calculations

A cross-docking study has been carried out by evaluating the best performing MMP structures in correctly relocate the cognate ligand of X-ray complexes.

To this aim highest resolution structures (<2.5 A) were retrieved from the PDB for MMP-2, MMP-8 and MMP-13 (Table S1).

MMP structures were prepared as reported in the Materials and methods section of the article.

Cognate ligands were extracted from the complexes; their structure has been fixed (bond order, hydrogen atoms, and protonation state). Ligands were submitted to minimization and conformational search such as reported for synthesized ligands.

Global minimum geometry of ligands was used for docking calculations in each MMP structure.

Highest ranking pose for each ligand in each receptor structure has been compared to the experimental one. Similarity to the experimental data has been evaluated by measuring the RMSD value.

RMSD calculated for all ligands are reported in Tables S2, S3 and S4 for, respectively MMP-2, MMP-8 and MMP-13.

Table S1. PDB-IDs of X-ray structures of MMP-2, MMP-8 and MMP-13 retrieved from PDB.

MMP-2	MMP-8	MMP-13
3AYU, 1HOV, 1QIB	1I76, 1KBC, 1MNC, 1ZP5, 1ZSO, 3DNG, 3DPE, 3DPF	1XUC, 2E2D, 2OZR, 3LJZ, 3WV3, 3ZXH, 3O2X, 4A7B, 456C, 1FLS, 3KRY, 1YOU, 2D1N, 3TVC

Table S2. RMSD values obtained aligning best docked pose to experimental geometry in MMP-2 structures.

Ligand/Receptor	1HOV0001	1HOV0002	1HOV0003	1HOV0004	1HOV0005	1HOV0006	1HOV0007
1HOV	2.897	15.3	1.78	9.1	1.61	1.68	7.44
Ligand/Receptor	1HOV0008	1HOV0009	1HOV0010	1HOV0011	3AYU	1QIB	
1HOV	4.79	1.68	1.89	2.56	5.39	4.29	

Table S3. RMSD values obtained aligning best docked pose to experimental geometry in MMP-8 structures.

Ligand/Receptor	1I76	1MNC	1KBC	1KBC_B	1ZP5	1ZSO	3DNG	3DPE	3DPF
1I76	2.93	2.46	2.77	2.7	2.82	2.91	3.11	2.82	3.36
1MNC	0.61	0.57	0.68	2.21	0.63	6.62	0.42	0.68	0.87
1KBC	1.7	1.87	0.63	1.78	1.75	1.99	1.7	1.56	1.55
1ZP5	1.51	2.41	1.84	1.73	2.15	2.66	1.11	1.66	2.08
3DNG	15.59	15.5	15.35	15.6	14.7	14.5	0.91	15.41	1.44
3DPE	14.4	13.15	13.3	11.6	13	12.9	14.7	14	12.4
3DPF	14.7	11.9	14.9	14.3	13.6	13.9	2.1	1.56	1.84

Table S4. RMSD values obtained aligning best docked pose to experimental geometry in MMP-13 structures.

Ligand/ Receptor	1FLS	1XUC	1XUC_B	1YOU	2D1N	2E2D	2OZR	2OZR_F	3O2X	3O2X_B	3O2X_C
4JP4	7.26	8.52	3.06	2.24	2.33	3.97	3.4	3.07	2.86	2.25	3.35
4JPA	8	7.03	1.77	13.4	13.6	7.45	13.6	1.72	2.27	14.2	12.8
2OW9	14.7	10.2	15.3	13.6	15.1	14.7	1.96	0.7	16.6	14.2	16.5
456C	1.88	1.47	1.19	1.34	1.44	1.81	1.14	1.53	1.4	1.32	1.58
1XUC	15.6	12.1	13.4	14.93	13.4	16.3	16.2	3.65	14.4	13.4	14.4
1XUD	15.8	14	15.5	15.9	8.88	16.9	3.9	2.36	15.21	12	14.9
1XUR	15.8	0.39	0.53	15.66	14.4	7.23	2.03	1.45	2.38	14.7	15.3
1YOU	6.88	17.2	7.82	4.98	9.05	7.38	3.13	18.1	8.84	7.77	8.71
1ZTQ	12.29	1.12	1.66	1.74	0.93	3.38	1.46	1.72	1.51	1.74	1.78
2OZR	14.56	4.82	14.7	14.1	14.5	15.4	1.21	4.71	5.1	14.4	17.3
3ELM	8.28	4.67	4.34	1.01	3.81	4.95	8.04	1.14	5.34	6.31	3.93
3I7G	8.52	5.36	6.75	4.8	14.4	1.14	1.13	1.31	13.1	1.1	8.79
3I7I	11.1	1.69	1.27	6.53	14.4	1.86	1.63	1.6	6.79	1.6	1.52
3KEJ	14.7	11.02	2.12	11.7	11.9	16.4	2.6	2.1	11.8	11.6	14.7
3KEK	15	1.64	2.43	16.5	11.6	11.6	17.1	1.16	11.7	10	12
3KRY	3.68	2.55	2.37	2.04	1.68	2.32	3.6	2.35	1.6	2.5	9.11
3LJZ	7.93	2.59	2.16	2.77	2.51	2.03	2.95	2.28	7.43	2.23	6.46
3TVC	3.96	2.16	2.35	1.91	2.3	2.11	2.09	2.53	3.05	2.28	2.59
3WV3	11.4	1.86	11.2	12.5	9.66	9.74	2.95	0.66	0.7	1.03	11.3
3ZXH	3.65	2.52	5.54	0.57	5.7	2.54	4.43	5.46	2.95	2.03	2.16
4JP4	2.77	2.63	3.52	4.4	1.95	3.5	5.36	3.65	3.14	3.09	4JP4
4JPA	1.84	13.6	2.31	13.4	13.6	13.4	3.57	7.35	1.75	1.82	4JPA
2OW9	14.7	15.4	16.3	14.7	14.2	14.6	13.3	15.6	15.1	14.9	2OW9
456C	12.4	11.8	1.47	1.43	1.24	1.42	3.31	7.16	2.36	1.53	456C
1XUC	13.6	13.3	15.1	13.9	13.7	13.8	9.79	13.6	12.6	14.9	1XUC
1XUD	12.1	12	12.2	16.6	15.5	16.6	11.6	12	11.9	15.3	1XUD
1XUR	14.7	13.8	15.3	12	15.4	11.23	4.82	15.1	14.7	15.1	1XUR
1YOU	3.54	3.6	3.27	6.82	8.77	6.83	4.79	8.08	4.88	3.21	1YOU
1ZTQ	2.61	2.6	1.88	1.33	2.26	2.92	2.73	3.25	1.03	1.02	1ZTQ
2OZR	14.5	14.7	15.9	16.2	14.6	14.5	14.1	15.4	14.82	15.3	2OZR
3ELM	7.7	7.34	7.5	1.46	3.29	1.18	7.7	7.43	6.77	2.81	3ELM
3I7G	1.09	12.5	4.48	4.78	1.1	5.77	13.8	1.14	5.15	1.22	3I7G
3I7I	1.64	1.64	13.5	6.96	6.23	5.14	1.54	2.2	2.02	1.7	3I7I
3KEJ	12	11.6	11.8	16.2	12	11.9	15.1	16	1.12	11	3KEJ
3KEK	11.5	12.2	17.4	18.3	11.5	11.8	14.6	16.7	17.4	11.8	3KEK
3KRY	4.6	2.08	1.68	9.19	2.51	7.9	8.51	9.5	2.1	2.64	3KRY
3LJZ	2.46	2.86	1.91	1.6	2.43	2.19	2.25	1.31	2.2	1.86	3LJZ
3TVC	2.5	2.28	2.22	2.37	2.27	2.37	3.93	5.11	2.12	2.43	3TVC
3WV3	1.19	9.86	9.8	11.2	2.95	11.3	4.28	5.04	4.46	0.43	3WV3
3ZXH	2.41	6.48	5.66	2.86	5.57	2.86	4.5	3.18	2.3	2.28	3ZXH

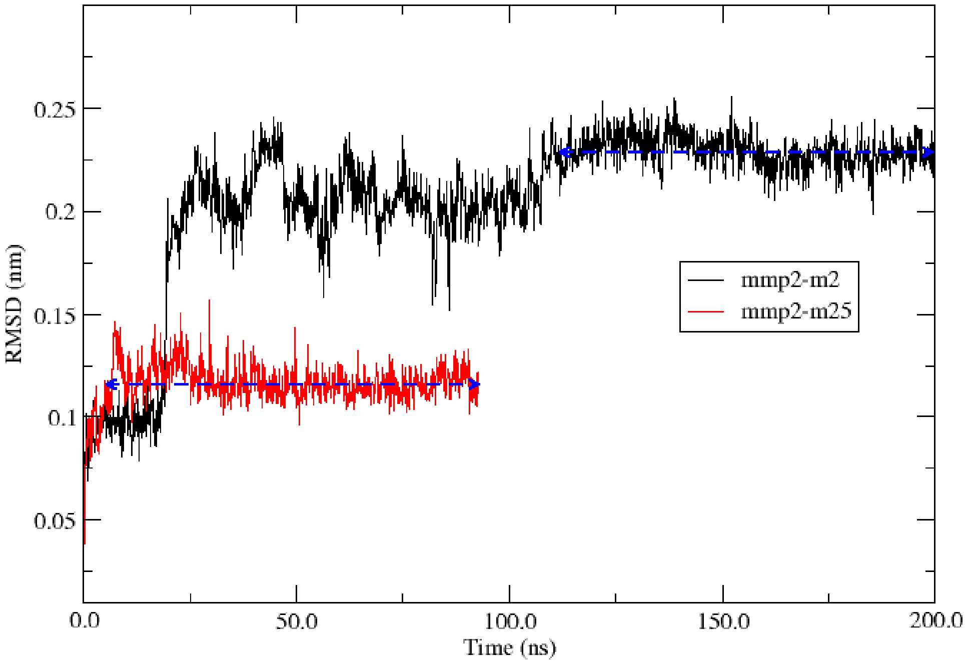

Figure S1. Root mean squared deviations of the backbone atom positions for the **1g** (black) and **1h** (red) bound complexes with MMP-2. Dashed arrows evidence stable segments of trajectory (last 90 ns).

Chemistry

***N*-Benzyl-*N*'-quinolin-3-ylurea (1a).** White solid (56% yield); DCM/MeOH 95:5 for chromatography; m.p. 206–208 °C; ^{1}H-NMR (DMSO*d*$_6$) δ 4.34 (d, 2H, *J* = 6.0 Hz, CH$_2$NH), 6.88 (t, 1H, *J* = 5.7 Hz, NHCH$_2$), 7.21–7.32 (m, 1H, CHAr), 7.32 (d, 3H, *J* = 4.5 Hz, CHAr), 7.46–7.56 (m, 2H, CHAr), 7.81 (dd, 1H, *J* = 6.0 Hz, *J* = 1.5 Hz, CHAr), 7.88 (dd, 1H, *J* = 6.0 Hz, *J* = 1.5 Hz, CHAr), 7.96 (d, 1H, *J* = 2.7 Hz, CHAr), 8.76 (d, 1H, *J* = 2.7 Hz, CHAr), 9.09 (s, 1H, NHCO); ^{13}C-NMR (DMSO*d*$_6$) δ 43.5, 127.4, 127.4, 127.5, 127.8, 127.8, 128.9, 129.0, 129.1, 134.9, 140.8, 143.9, 144.8, 155.9.

***N*-Benzyl-*N*′-quinolin-4-ylurea (1b).** Yellowish solid (51% yield); DCM/MeOH 95:5 for chromatography; m.p. 167–169 °C; ^{1}H-NMR (DMSO*d*$_6$) δ 4.38 (d, 2H, *J* = 5.4 Hz, CH$_2$NH), 7.23–7.29 (m, 1H, NHCH$_2$), 7.34–7.43 (m, 5H, CHAr), 7.56–7.74 (m, 2H, CHAr), 7.92 (d, 1H, *J* = 8.1 Hz, CHAr), 8.16 (d, 1H, *J* = 8.1 Hz, CHAr), 8.21 (d, 1H, *J* = 5.4 Hz, CHAr), 8.64 (d, 1H, *J* = 5.4 Hz, CHAr), 9.15 (bs, 1H, NH$_{Ar}$); ^{13}C-NMR (DMSO*d*$_6$) δ 43.3, 107.7, 119.6, 121.3, 125.9, 127.4, 127.8, 128.9, 129.6, 130.0, 140.0, 143.3, 148.8, 151.3, 155.0.

***N*-Benzyl-*N*′-quinolin-5-ylurea (1c).** White-yellowish needles (46% yield); recrystallized from MeOH; m.p. 251 °C (dec); ^{1}H-NMR (DMSO*d*$_6$) δ 4.34 (d, 2H, *J* = 5.7 Hz, CH$_2$NH), 7.01 (t, 1H, *J* = 5.7 Hz, NHCH$_2$), 7.22–7.26 (m, 1H, CHAr), 7.33 (d, 4H, *J* = 5.7 Hz, CHAr), 7.51–7.55 (d, 1H, *J* = 8.5 Hz, CHAr), 7.64 (d, 2H, *J* = 4.4 Hz, CHAr), 8.03–8.05 (m, 1H, CHAr), 8.46–8.49 (dd, 1H, *J* = 8.5 Hz, J = 1.4 Hz, CHAr), 8.76 (bs, 1H, NHPh), 8.86–8.87 (dd, 1H, *J* = 4.1 Hz, *J* = 1.6 Hz, CHAr); ^{13}C-NMR (DMSO*d*$_6$) δ 43.6, 117.1, 121.1, 121.3, 123.7, 127.5, 127.9, 129.0, 130.1, 130.7, 136.2, 140.7, 148.8, 150.8, 156.1.

***N*-Benzyl-*N*′-quinolin-8-ylurea (1d).** White solid (54% yield); DCM/MeOH 95:5 for chromatography; ^{1}H-NMR (CDCl$_3$): δ 4.49 (s, 2H, CH2); 7.24–7.43 (m, 7H, CHAr); 7.52 (t, *J* = 6.0 Hz, 1H, CHAr); 8.14 (dd, *J* = 6.3 Hz, *J* = 1.8 Hz, 1H, CHAr); 8.57 (dd, *J* = 6.6 Hz, *J* = 1.5 Hz, 1H, CHAr); 8.70 (dd, *J* = 2.4 Hz, *J* = 1.8 Hz, 1H, CHAr); 9.04 (s, 1H, NH); ^{13}C-NMR (CDCl$_3$): δ 43.8, 118.4, 120.5, 126.1, 126.2, 126.6, 128.3, 128.4, 128.4, 128.6, 129.9, 141.7, 141.9, 150.5, 173.6.

***N*-Benzyl-*N*′-isoquinolin-1-ylurea (1e).** Yellow solid (67% yield); DCM/MeOH 95:5 for chromatography; m.p. 164–165 °C; ^{1}H-NMR (CDCl$_3$) δ 4.67 (s, 2H, CH$_2$), 7.22–7.33 (m, 3H, CHAr),

7.35 (t, 2H, *J* = 7.2 Hz, CHAr), 7.43 (d, 2H, *J* = 7.5 Hz, CHAr), 7.52 (t, 1H, *J* = 7.8 Hz, CHAr), 7.68 (t, 1H, *J* = 6.9 Hz, CHAr), 7.76 (d, 1H, *J* = 8.1 Hz, CHAr), 7.99 (d, 1H, *J* = 6.0 Hz, CHAr), 8.19 (s, 1H, NH), 10.66 (s, 1H, NHPh); ^{13}C-NMR ($CDCl_3$) δ 43.8, 118.4, 120.5, 126.1, 126.2, 126.5, 128.2, 128.4, 128.4, 128.6, 129.9, 141.7, 141.8, 150.5, 154.9.

***N*-Benzyl-*N*′-isoquinolin-3-ylurea (1f).** Yellow solid (59% yield); cyclohexane/EtOAc 1:1 for chromatography; m.p. 168–170 °C; ^{1}H-NMR (CD_3OD) δ 4.51 (s, 2H, CH_2), 7.24–7.46 (m, 6H, CHAr), 7.60–7.66 (m, 1H, CHAr), 7.74 (d, 1H, *J* = 8.4 Hz, CHAr), 7.93 (d, 1H, *J* = 8.7 Hz, CHAr), 8.96 (s, 1H, CHAr); ^{13}C-NMR (CD_3OD) δ 43.2, 104.7, 125.1, 125.5, 125.7, 126.9, 127.0, 127.6, 128.4, 131.0, 150.3.

***N*-Benzyl-*N*′-isoquinolin-4-ylurea (1g).** White solid (56% yield); cyclohexane/EtOAc 7:3 for chromatography; m.p. 235–237 °C; ^{1}H-NMR (CD_3OD) δ 4.44 (s, 1H, CH_2), 7.34–7.36 (m, 5H, CHAr), 7.70 (t, 1H, *J* = 6.9 Hz, CHAr), 7.81 (t, 1H, *J* = 6.9 Hz, CHAr), 8.04 (d, 1H, *J* = 8.1 Hz, CHAr), 8.10 (d, 1H, *J* = 8.1 Hz, CHAr), 8.81 (s, 1H, CHAr), 8.98 (s, 1H, CHAr); ^{13}C-NMR (CD_3OD) δ 43.6, 121.0, 127.0, 127.7, 128.1, 128.4, 129.2, 130.5, 130.9, 133.9, 135.5, 147.5, 157.2.

***N*-Benzyl-*N*′-isoquinolin-5-ylurea (1h).** Yellowish solid (66% yield); recrystallized from MeOH; m.p. 248–249 °C; ^{1}H-NMR (DMSO*d*$_6$) δ 4.35 (d, 2H, *J* = 5.7 Hz, CH_2NH), 7.06 (t, 1H, *J* = 5.7 Hz, NHCH_2), 7.20–7.34 (m, 5H, CHAr), 7.58 (d, 1H, *J* = 7.8 Hz, CHAr), 7.71 (d, 1H, *J* = 7.8 Hz, CHAr), 7.91 (d, 1H, *J* = 6.0 Hz, CHAr), 8.29 (d, 1H, *J* = 7.8 Hz, CHAr), 8.51 (d, 1H, *J* = 6.0 Hz, CHAr), 8.74 (bs, 1H, NHAr), 9.24 (s, 1H, CHAr); ^{13}C-NMR (DMSO*d*$_6$) δ 43.6, 115.0, 119.9, 121.8, 127.5, 127.9, 128.1, 128.2, 129.0, 129.4, 135.2, 140.6, 143.1, 153.3, 156.0.

***N*-Benzyl-*N*′-isoquinolin-8-ylurea (1i).** Yellowish solid (49% yield); DCM/MeOH 95:5 for chromatography; m.p. 206–208 °C; ^{1}H-NMR (DMSO*d*$_6$) δ 4.36 (d, 2H, *J* = 5.4 Hz, CH_2NH), 7.08 (t, 1H, *J* = 5.4 Hz, NHCH_2), 7.22–7.35 (m, 5H, CHAr), 7.54 (d, 1H, *J* = 7.8 Hz, CHAr), 7.65 (t, 1H, *J* = 7.5 Hz, CHAr) 7.76 (d, 1H, *J* = 5.7 Hz, CHAr), 8.14 (d, 1H, *J* = 7.5 Hz, CHAr), 8.47 (d, 1H, *J* = 5.7 Hz, CHAr), 9.01 (s, 1H, CHAr), 9.49 (bs, 1H, NH_{Ar}); ^{13}C-NMR (DMSO*d*$_6$) δ 43.4, 117.3, 120.6, 120.8, 120.9, 127.3, 127.6, 128.8, 131.3, 136.4, 136.6, 140.4, 143.1, 147.1, 155.7.

***N*-Benzyl-*N*′-1*H*-indol-5-ylurea (1j).** Brown solid (53% yield); recrystallized from EtOAc; m.p. 197–198 °C; ^{1}H-NMR (CD_3OD) δ 4.37 (s, 2H, CH_2NH), 6.37 (d, 1H, *J* = 3.0 Hz, CHAr), 7.00–7.03 (dd, 1H, *J* = 8.7 Hz, *J* = 2.2 Hz, CHAr), 7.18–7.32 (m, 7H, CHAr), 7.51 (d, 1H, *J* = 1.6 Hz, CHAr); ^{13}C-NMR (CD_3OD) δ 41.8, 99.5, 109.4, 111.5, 115.3, 123.6, 125.2, 125.4, 126.7, 126.9, 129.0, 132.1, 138.5, 156.7.

***N*-Benzyl-*N*′-1*H*-indol-6-ylurea (1k).** White solid (42% yield); recrystallized from MeOH; m.p. 211–213 °C; ^{1}H-NMR (DMSO*d*$_6$) δ 4.28 (d, 2H, *J* = 6.0 Hz, C*H*$_2$NH), 6.27 (s, 1H, CHAr), 6.47 (t, 1H, *J* = 5.7 Hz, NHCH_2), 6.75 (d, 1H, *J* = 8.1 Hz, CHAr), 7.15 (s, 1H, CHAr), 7.21–7.34 (m, 5H, CHAr), 7.73 (s, 1H, CHAr), 8.38 (s, 1H, NH), 10.83 (s, 1H, CHAr); ^{13}C-NMR (DMSO*d*$_6$) δ 43.4, 101.2, 101.4, 112.3, 120.4, 123.7, 124.7, 127.3, 127.8, 128.9, 135.2, 136.9, 141.2, 156.2.

***N*-Benzyl-*N*′-1*H*-indazol-5-ylurea (1l).** Pink solid (59% yield); recrystallized from petroleum ether/$CHCl_3$; m.p. 242 °C (dec); ^{1}H-NMR (DMSO*d*$_6$) δ 4.29 (d, 2H, *J* = 5.7 Hz, CH_2NH), 6.54 (t, 1H, *J* = 5.7 Hz, NHCH_2), 7.20–7.40 (m, 7H, CHAr), 7.84 (s, 1H, CHAr), 7.91 (s, 1H, CHAr), 8.47 (s, 1H, NHPh), 12.69 (s, 1H, NH indazole); ^{13}C-NMR (DMSO*d*$_6$) δ 43.4, 108.3, 110.7, 120.6, 123.6, 127.3, 127.8, 128.9, 133.6, 134.0, 136.8, 141.2, 156.3.

***N*-Benzyl-*N*′-1*H*-indazol-6-ylurea (1m).** Brown solid (52% yield); recrystallized from DCM; m.p. 198–199 °C; ^{1}H-NMR (DMSO*d*$_6$) δ 4.30 (d, 2H, *J* = 5.7 Hz, CH_2NH), 6.63 (t, 1H, *J* = 5.7 Hz, NHCH_2), 6.82 (dd, 1H, *J* = 8.7 Hz, *J* = 1.8 Hz, CHAr), 7.19–7.32 (m, 5H, CHAr), 7.55 (d, 1H, *J* = 8.7 Hz, CHAr), 7.87 (s, 1H, CHAr), 7.90 (s, 1H, CHAr), 8.72 (s, 1H, NHPh), 12.72 (s, 1H, NH indazole); ^{13}C-NMR (DMSO*d*$_6$) δ 43.4, 97.2, 114.1, 118.6, 121.1, 127.4, 127.8, 129.0, 133.8, 139.4, 140.9, 141.5, 156.0.

***N*-Benzyl-*N*′-1-naphthylurea (1n).** White solid (79 % yield); recrystallized from MeOH; m.p. 196–197 °C; ^{1}H-NMR (DMSO*d*$_6$) δ 4.37 (d, 2H, *J* = 5.7 Hz, CH_2NH), 7.06 (t, 1H, *J* = 5.7 Hz, NHCH_2), 7.22–7.56 (m, 9H, CHAr), 7.86–7.89 (m, 1H, CHAr), 8.02–8.10 (m, 2H, CHAr), 8.64 (s, 1H, NH);

^{13}C-NMR (DMSO*d*$_6$) δ 43.4, 116.9, 121.8, 122.6, 125.9, 126.2, 126.4, 127.3, 127.7, 127.7, 128.8, 128.9, 134.1, 135.5, 140.6, 156.1.

***N*-Benzyl-*N*′-2-naphthylurea (1o).** White solid (48% yield); recrystallized from MeOH; m.p. 208–210 °C; ^{1}H-NMR (DMSO*d*$_6$) δ 4.32 (d, 2H, *J* = 5.7 Hz, CH_2NH), 6.71 (t, 1H, *J* = 5.7 $NHCH_2$), 7.45–7.20 (m, 8H, CHAr), 7.70–7.78 (m, 3H, CHAr), 8.04 (s, 1H, CHAr), 8.78 (s, 1H, NH); ^{13}C-NMR (DMSO*d*$_6$) δ 43.0, 113.2, 120.1, 124.2, 126.9, 127.4, 127.4, 127.8, 128.0, 128.9, 129.0, 129.4, 134.4, 138.8, 141.0, 155.9.

***N*-Benzyl-*N*′-pyridin-2-ylurea (1p).** Yellow solid (54% yield); cyclohexane/EtOAc 1:1 for chromatography; m.p. 148–150 °C; ^{1}H-NMR ($CDCl_3$) δ 6.62 (d, 2H, *J* = 5.7 Hz, CH_2), 6.82–6.86 (m, 2H, CHAr), 7.25–7.39 (m, 5H, CHAr), 7.53–7.58 (m, 1H, CHAr), 8.10–8.12 (m, 1H, CHAr), 9.00 (s, 1H, NH), 9.80 (s, 1H, NH); ^{13}C-NMR ($CDCl_3$) δ 43.9, 112.3, 116.9, 127.2, 127.5, 127.6, 128.7, 138.6, 139.5, 146.0, 153.5, 159.6.

***N*-Benzyl-*N*′-pyridin-3-ylurea (1q).** White solid (56% yield); $CHCl_3$/acetone 7:3 for chromatography; m.p. 154–156 °C; ^{1}H-NMR ($CDCl_3$) δ 6.62 (d, 2H, *J* = 5.7 Hz, CH_2), 6.82–6.86 (m, 2H, CHAr), 7.25–7.39 (m, 5H, CHAr), 7.53–7.58 (m, 1H, CHAr), 8.10–8.12 (m, 1H, CHAr), 9.00 (s, 1H, NH), 9.80 (s, 1H, NH); ^{13}C-NMR ($CDCl_3$) δ 44.2, 122.0, 127.0, 127.6, 128.2, 135.7, 139.7, 140.3, 141.8, 156.1.

***N*-Benzyl-*N*′-phenylurea (1r).** White solid (82% yield); recrystallized from MeOH; m.p. 169–170 °C; ^{1}H-NMR (DMSO*d*$_6$) δ 4.28 (d, 2H, *J* = 6.0 Hz, CH_2), 6.59 (t, 1H, *J* = 6.0 Hz, $NHCH_2$), 6.84–6.90 (m, 1H, CHAr), 7.17–7.40 (m, 9H, CHAr), 8.54 (s, 1H, NH); ^{13}C-NMR (DMSO*d*$_6$) δ 43.4, 118.3, 121.8, 127.4, 127.8, 129.0, 129.4, 141.0, 141.1, 155.9.

***N*-Benzyl-*N*′-(4-hydroxyphenyl)urea (1s).** White solid (99% yield); recrystallized from DCM; m.p. 177–179 °C; ^{1}H-NMR (DMSO*d*$_6$) δ 4.24 (d, 2H, *J* = 5.7 Hz, CH_2NH), 6.40 (t, 1H, *J* = 5.7 Hz, NH), 6.60 (d, 2H, *J* = 9.0 Hz, CHAr), 7.13 (d, 2H, *J* = 8.7 Hz, CHAr), 7.20–7.32 (m, 5H, CHAr), 8.14 (s, 1H, NH), 8.90 (s, 1H, OH); ^{13}C-NMR (DMSO*d*$_6$) δ 43.4, 110.5, 102.1, 120.6, 120.7, 120.8, 130.2, 140.1, 150.2, 150.6.

***N*-Benzyl-*N*′-(2,3-dimethoxybenzyl)urea (1t).** White solid (95% yield); recrystallized from MeOH; m.p. 149°C ^{1}H-NMR (DMSO*d*$_6$) δ 3.70 (s, 3H, OCH_3), 3.77 (s, 3H, OCH_3), 4.20 (d, 2H, *J* = 5.7 Hz, CH_2NH), 4.21 (d, 2H, *J* = 6.0 Hz, CH_2NHPh), 6.27 (t, 1H, *J* = 5.7 Hz, $NHCH_2$), 6.44 (t, 1H, *J* = 6.0 Hz, $NHCH_2Ph$), 6.78–6.81 (m, 1H, CHAr), 6.89–7.02 (m, 2H, CHAr), 7.19–7.31 (m, 5H, CHAr); ^{13}C-NMR (DMSO*d*$_6$) δ 38.6, 43.6, 56.3, 60.7, 112.1, 120.7, 124.4, 127.2, 127.7, 128.9, 134.7, 141.6, 146.8, 152.9, 158.7.

***N*-Benzyl-*N*′-(3,4-dimethoxybenzyl)urea (1u).** White solid (97% yield); recrystallized from MeOH; m.p. 127°C; ^{1}H-NMR (DMSO*d*$_6$) δ 3.69 (s, 3H, OCH_3), 3.70 (s, 3H, OCH_3), 4.14 (d, 2H, *J* = 5.7 Hz, CH_2NH), 4.21 (d, 2H, *J* = 6.0 Hz, CH_2NHPh), 6.35 (t, 1H, *J* = 6.0 Hz, $NHCH_2$), 6.41 (t, 1H, *J* = 5.7 Hz, $NHCH_2Ph$), 6.73–6.77 (m, 1H, CHAr), 6.84–6.87 (m, 2H, CHAr), 7.17–7.31 (m, 5H, CHAr); ^{13}C-NMR (DMSO*d*$_6$) δ 43.4, 43.6, 56.0, 56.2, 111.6, 112.3, 119.7, 127.2, 127.6, 128.9, 134.0, 141.7, 148.2, 149.2, 158.7.

***N*-*N*'dibenzylurea (1v).** White solid (94% yield); recrystallized from MeOH; m.p. 170–171°C; ^{1}H-NMR (DMSO*d*$_6$) δ 4.21 (d, 4H, *J* = 6.0 Hz, 2 CH_2NH), 4.34 (t, 2H, *J* = 6.0 Hz, 2 $NHCH_2$), 7.16–7.33 (m, 10H, CHAr); ^{13}C-NMR (DMSO*d*$_6$) δ 43.6, 127.2, 127.7, 128.9, 141.7, 158.8.

***N*-Benzyl-*N*′-(1-phenylethyl)urea (1w).** White needles (82% yield); recrystallized from ciclohexane/DCM; m.p. 116–118 °C; ^{1}H-NMR (DMSO*d*$_6$) δ 1.30 (d, 3H, *J* = 7.2 Hz, CH_3), 4.17 (d, 2H, *J* = 5.7 Hz, CH_2NH), 4.73 (m, 1H, $CHCH_3$), 6.26 (t, 1H, *J* = 5.7 Hz, $NHCH_2$), 6.42 (d, 1H, *J* = 8.4 Hz, NHCH), 7.16–7.32 (m, 10H, CHAr); ^{13}C-NMR (DMSO*d*$_6$) δ 24.0, 43.4, 49.3, 126.4, 127.1, 127.2, 127.6, 128.8, 141.5, 146.4, 157.9.

***N*-Benzyl-*N*′-butylurea (1x).** White solid (63% yield); DCM/MeOH 95:5 for chromatography; m.p. 100–101 °C; ^{1}H-NMR (CD_3OD) δ 0.92 (t, 3H, *J* = 7.2 Hz, CH_3), 1.28–1.51 (m, 4H, $CH_3CH_2CH_2$),

3.12 (t, 2H, *J* = 7.2 Hz, CH_2CH_2NH), 4.29 (s, 2H, CH_2NH), 7.18–7.32 (m, 5H, CHAr); ^{13}C-NMR (CD_3OD) δ 12.9, 19.8, 32.3, 39.5, 43.5, 126.7, 126.9, 128.2, 140.2, 158.2.

***N*-Benzyl-*N*′-(1-benzylpiperidin-4-yl)urea (1y).** White solid (74% yield); recrystallized from ciclohexane/EtOAc; m.p. 114–116 °C ; ^{1}H-NMR ($CDCl_3$) δ 1.34–1.45 (m, 2H, CH_2 pip), 1.85–2.11 (m, 4H, CH_2 pip), 2.75 (m, 2H, CH_2 pip), 3.46 (s, 2H, CH_2N), 3.51–3.62 (m, 1H, CH pip), 4.31 (d, 2H, *J* = 5.7 Hz, CH_2NH), 4.47 (d, 1H, *J* = 8.1 Hz, NHCH), 4.87 (t, 1H, *J* = 5.7 Hz, $NHCH_2$), 7.20–7.33 (m, 10H, CHAr); ^{13}C-NMR ($CDCl_3$) δ 33.0, 44.7, 47.5, 52.5, 63.2, 127.3, 127.5, 127.6, 128.4, 128.8, 129.4, 138.3, 139.4, 157.7.

***N*-Benzyl-*N*′-pyrrolidin-1-ylurea (1z).** White solid (41% yield); DCM/MeOH 95:5 for chromatography; m.p. 124–126 °C; ^{1}H-NMR (DMSO*d*$_6$) δ 1.62–1.71 (m, 4H, CH_2 pyrr), 2.60–2.82 (m, 4H, CH_2 pyrr), 4.20 (d, 2H, *J* = 6.3 Hz, CH_2NH), 6.99 (t, 1H, *J* = 6.3 Hz, $NHCH_2$), 7.08 (bs, 1H, NHN), 7.16–7.30 (m, 5H, CHAr); ^{13}C-NMR (DMSO*d*$_6$) δ 22.4, 42.8, 55.4, 127.0, 127.5, 128.8, 141.8, 158.9.

***N*-Benzyl-4-methylpiperazine-1-carboxamide (1α).** White solid (55% yield); DCM/MeOH 95:5 for chromatography; m.p. 127–128 °C; ^{1}H-NMR (CD_3OD) δ 2.30 (s, 3H, CH_3), 2.42 (t, 4H, *J* = 5.4 Hz, CH_2 pip), 3.43 (t, 4H, *J* = 5.4 Hz, CH_2 pip), 4.34 (s, 2H, CH_2NH), 7.21–7.29 (m, 5H, CHAr); ^{13}C-NMR (CD_3OD) δ 43.2, 44.0, 44.9, 54.4, 126.6, 127.0, 128.1, 140.2, 158.8.

***N*-Isoquinolin-5-yl-2-phenylacetamide (2a).** White solid (55% yield); recrystallized from hexane/EtOAc; m.p. 186–188 °C; ^{1}H-NMR ($CDCl_3$) δ 3.91 (s, 2H, CH_2), 7.14 (d, 1H, *J* = 5.7 Hz, CHAr), 7.34–7.29 (m, 5H, CHAr), 7.60 (d, 1H, *J* = 8.1 Hz, CHAr), 7.78 (d, 1H, *J* = 8.4 Hz, CHAr), 8.07 (d, 1H, *J* = 7.5 Hz, CHAr), 8.35 (d, 1H, *J* = 5.4 Hz, CHAr), 9.18 (s, 1H, CHAr); ^{13}C-NMR ($CDCl_3$) δ 44.7, 118.4, 120.5, 126.1, 126.2, 126.5, 128.2, 128.4, 128.4, 128.6, 129.9, 141.7, 141.8, 150.5, 173.6.

***N*-Isoquinolin-5-yl-3-phenylpropanamide (2b).** White solid (63% yield); recrystallized from hexane/EtOAc; m.p. 133–135 °C; ^{1}H-NMR ($CDCl_3$) δ 2.87 (t, 2H, *J* = 6.0 Hz, CH_2CO), 3.13 (t, 2H, *J* = 7.0 Hz, CH_2Ar), 7.14 (d, 1H, *J* = 5.7 Hz, CHAr), 7.34–7.29 (m, 5H, CHAr), 7.60 (d, 1H, *J* = 8.1 Hz, CHAr), 7.78 (d, 1H, *J* = 8.4 Hz, CHAr), 8.07 (d, 1H, *J* = 7.5 Hz, CHAr), 8.35 (d, 1H, *J* = 5.4 Hz, CHAr), 9.18 (s, 1H, CHAr); ^{13}C-NMR ($CDCl_3$) δ 34.6, 35.8, 118.4, 120.5, 126.1, 126.2, 126.5, 128.2, 128.4, 128.4, 128.6, 129.9, 141.7, 141.8, 150.5, 173.6.

***N*-(Phenylmethyl)isoquinoline-5-carboxamide (2c).** White solid (70% yield); DCM/MeOH 95:5 for chromatography; m.p. 182–183 °C; ^{1}H-NMR ($CDCl_3$) δ 4.71 (d, 2H, *J* = 6.0 Hz, CH_2NH), 6.58 (t, 1H, *J* = 6.0 Hz, $NHCH_2$), 7.32–7.42 (m, 5H, CHAr), 7.58 (t, 1H, *J* = 6.9 Hz, CHAr), 7.87 (dd, 1H, J = 7.2 Hz, *J* = 1.2 Hz, CHAr), 8.03 (d, 1H, *J* = 8.1 Hz, CHAr), 8.20 (d, 1H, *J* = 6.0 Hz, CHAr), 8.50 (d, 1H, *J* = 6.6 Hz, CHAr), 9.20 (s, 1H, CHAr); ^{13}C-NMR ($CDCl_3$) δ 44.2, 118.5, 126.4, 127.8, 127.9, 128.3, 128.9, 129.5, 130.6, 132.9, 133.2, 137.7, 143.3, 152.2, 167.7.

***N*-(2-Phenylethyl)isoquinoline-5-carboxamide (2d).** White solid (58% yield); DCM/MeOH 95:5 for chromatography; m.p. 120–121 °C; ^{1}H-NMR ($CDCl_3$) δ 2.99 (t, 2H, *J* = 7.2 Hz, CH_2Ph), 3.80 (ql, 2H, CH_2NH), 6.33 (t, 1H, *J* = 6.9 Hz, $NHCH_2$), 7.22–7.35 (m, 5H, CHAr), 7.51 (t, 1H, *J* = 6.9 Hz, CHAr), 7.69 (dd, 1H, *J* = 6.9 Hz, *J* = 0.9 Hz, CHAr), 7.95 (s, 1H, CHAr), 7.98 (d, 1H, *J* = 6.0 Hz, CHAr), 8.44 (d, 1H, *J* = 6.0 Hz, CHAr), 9.15 (s, 1H, CHAr); ^{13}C-NMR ($CDCl_3$) δ 35.5, 41.1, 118.2, 126.3, 126.7, 128.5, 128.7, 128.8, 129.2, 130.3, 132.9, 133.2, 137.8, 143.6, 152.4, 168.0.

***N*-Isoquinolin-6-ylbenzenesulfonamide (2e).** Yellow solid (61% yield); m.p. 228–230 °C; ^{1}H-NMR ($CDCl_3$) δ 7.39 (s, 1H, NHCHAr), 7.43 (d, 2H, *J* = 7.5 Hz, CHAr), 7.57 (m, 3H, CHAr), 7.66 (d, 2H, *J* = 7.2 Hz, CHAr), 7.80 (d, 1H, *J* = 6.3 Hz, CHAr), 7.99 (d, 1H, *J* = 7.8 Hz, CHAr), 8.30 (d, 1H, *J* = 6.0 Hz, CHAr), 9.20 (s, 1H, CHAr); ^{13}C-NMR ($CDCl_3$) δ 116.0, 116.9, 118.4, 123.8, 127.0, 129.2, 129.6, 133.1, 134.7, 140.5, 142.2, 143.1, 153.6.

1,1-Dimethylethyl [(4-aminophenyl)methyl]carbamate (3). Di(terz-butyl)dicarbonate (1.1 eq) was added to a solution of 4-aminobenzylamine (1 eq) in THF (10 mL), and the mixture was stirred at room temperature. After 2 h, the solvent was evaporated under reduced pressure to give, the crude material that was purified by column chromatography, using DCM as eluent. Yellowish solid

(66% yield); m.p. 72–74 °C; ^{1}H-NMR ($CDCl_3$) δ 1.47 (s, 9H, CH_3), 3.54 (bs, 2H, NH_2), 4.18 (d, 2H, *J* = 5.1 Hz, CH_2NH), 4.74 (bs, 1H, NH), 6.66 (d, 2H, *J* = 6.9 Hz, CHAr), 7.07 (d, 2H, *J* = 6.9 Hz, CHAr); ^{13}C-NMR ($CDCl_3$) δ 28.4, 44.3, 115.3, 128.8, 145.2, 152.2, 155.8.

1,1-Dimethylethyl ({4-[(phenylsulfonyl)amino]phenyl}methyl)carbamate (4). Pink solid (86% yield); DCM/MeOH 95:5 for chromatography; m.p. 160–161 °C; ^{1}H-NMR ($CDCl_3$) δ 1.43 (s, 9H, CH_3), 4.21 (bs, 2H, CH_2), 4.83 (bs, 1H NH), 7.00–7.13 (m, 5H, 4 CHAr and NH), 7.39–7.78 (m, 5H, CHAr); ^{13}C-NMR ($CDCl_3$) δ 28.5, 44.0, 121.9, 127.1, 128.3, 129.0, 133.0, 135.4, 138.9, 147.4, 155.9.

1,1-Dimethylethyl ({4-[(phenylcarbonyl)amino]phenyl}methyl)carbamate (6). White solid (72% yield); DCM/MeOH 95:5 for chromatography; m.p. 169–171 °C; ^{1}H-NMR (CD_3OD) δ 1.45 (s, 9H, CH_3), 4.20 (bs, 2H, CH_2), 7.27 (d, 2H, *J* = 8.7 Hz, CHAr), 7.42–7.59 (m, 3H, CHAr), 7.64 (d, 2H, *J* = 8.7 Hz, CHAr), 7.90–7.93 (m, 2H CHAr); ^{13}C-NMR (CD_3OD) δ 27.3, 43.2, 120.9, 127.1, 127.2, 128.2, 131.4, 127.9, 134.8, 137.3, 155.5, 165.3.

***N*-[4-({[(Isoquinolin-5-ylamino)carbonyl]amino}methyl)phenyl] benzensulfonamide (5).** Pink crystals (45% yield); recrystallized from DCM/MeOH; m.p. 117–119 °C; ^{1}H-NMR (DMSOd_6) δ 4.05 (d, 2H, *J* = 5.7 Hz, CH_2NH), 6.28 (t, 1H, *J* = 5.7 Hz, $NHCH_2$), 6.97–7.11 (m, 5H, CHAr), 7.49–7.74 (m, 10H, CHAr and NH), 9.96 (bs, 1H, NH), 10.22 (s, 1H, CHAr); ^{13}C-NMR (DMSOd_6) δ 42.7, 110.0, 112.1, 120.6, 127.1, 128.2, 128.3, 129.6, 133.3, 136.4, 137.1, 139.9, 136.5, 141.5, 143.6, 144.6, 158.3.

***N*-[4-({[(Isoquinolin-5-ylamino)carbonyl]amino}methyl)phenyl]benzamide (7).** Yellowish crystals (48% yield); recrystallized from ciclohexane/EtOH; m.p. 200–202 °C; ^{1}H-NMR (DMSOd_6) δ 4.33 (d, 2H, *J* = 5.4 Hz, CH_2NH), 7.19 (t, 1H, *J* = 5.4 Hz, $NHCH_2$), 7.31–7.95 (m, 11H, CHAr), 8.00 (d, 1H, *J* = 6.6 Hz, CHAr), 8.31 (d, 1H, *J* = 7.5 Hz, CHAr), 8.51 (d, 1H, *J* = 5.1 Hz, CHAr), 8.88 (bs, 1H, NH), 9.25 (bs, 1H, NH), 10.24 (s, 1H, CHAr); ^{13}C-NMR (DMSOd_6) δ 42.8, 114.9, 119.6, 120.8, 121.5, 128.0, 128.1, 128.8, 130.3, 131.9, 137.1, 138.4, 139.5, 142.7, 146.9, 153.0, 155.8, 165.8.
